# Supplementary material for: A qualitative study of perceived barriers and facilitators to point-of-care ultrasound use among Veterans Affairs Emergency Department providers
Source: PLoS One. 2024 Nov 7;19(11):e0310404. doi: 10.1371/journal.pone.0310404 (PMC11542812; doi:10.1371/journal.pone.0310404)
Supplement: S1 Appendix — (DOCX) [file pone.0310404.s001.docx]

Semi-structured interview questions – summer 2022

Each recorded audio interview will begin with a clear statement of verbal consent as below:

*The following interview will be recorded as part of our study, “Implementation of a point-of-care ultrasound training program for emergency department providers in the [name redacted] Healthcare system”. The principal investigator for this study is [name redacted]. You may direct additional questions to the study PI. Identifying data will not be shared with parties outside of the study team. You will not directly benefit from this study but may help future ultrasound experiences at the [name redacted] Healthcare System. Do you consent with participating in this recorded interview?*

**Barriers and Facilitators to Point-of-Care Ultrasound Use in the [name redacted] VA Emergency Department**

*We will begin with some introductory questions regarding your clinical training background.*

1. Which type of residency training did you complete? (e.g. emergency medicine, internal medicine, family medicine, etc.)
2. Did you complete a fellowship and if so, in what?
3. Do you practice at another community or academic site? Which type?

***The following questions ask about clinical ultrasound use and point-of-care ultrasound (POCUS) skills.***

1. Why do you think POCUS would be useful in general in the emergency department?
2. How often do you personally use POCUS? (e.g. number of times per shift, number of times per month)
3. How do you incorporate point-of-care ultrasound into your clinical practice in the ED?
   1. Can you give an example of how you would use ultrasound for a procedure? (e.g. IV placement, central lines, paracentesis, thoracentesis, etc.)
   2. Can you give an example of how you would use ultrasound for diagnostic purposes? (e.g. cardiac, lung, abdominal exams, soft tissue, MSK, FAST exam in trauma)
4. What are the most common exams for which you use POCUS? (e.g. Cardiac, IVC for volume status, lung, aorta, renal, bladder volume, DVT, biliary, soft tissue, MSK, ocular, pregnancy/gynecology, FAST exam/trauma, procedural guidance)

**Ultrasound Education/POCUS knowledge:**

1. Which ultrasound exams would be most useful for you to practice more (e.g. already know how to do but would appreciate more tips or teaching about)?
2. Which ultrasound exams would be most useful for you to learn (e.g. new application that you don’t already know)?
3. Do you believe that it is important for residents to learn ultrasound, and why?
4. How comfortable do you feel teaching residents or other providers about ultrasound?

*We will now transition to key questions regarding barriers and facilitators of POCUS use.*

1. ***We are aiming to identify barriers that prevent you from using ultrasound in the ED*. (Inner setting)**
2. **Equipment management and upkeep:**
   1. The *[name redacted]* ED has two diagnostic Sonosite X-Portes and a vascular Nanomaxx ultrasound machine. Is this equipment adequate for clinical use? For teaching? Are the machines easy to locate and store? How is the image quality and software? Are the machines well upkept, or are they often missing or out-of-service?
   2. Do you have concerns about knowing how to manually operate the ultrasound machines? If so, what could help with this?
3. **Individual POCUS knowledge and skills:**
   1. What is your comfort level regarding your background ultrasound knowledge for obtaining diagnostic images? What about for procedures?
   2. How do you feel about your image interpretation skills when reviewing images?
   3. What could be done to improve your POCUS knowledge?
4. **Archiving (and billing) processes:**
   1. Do you feel that you do not have enough time while working to perform an ultrasound exam and document it? What could improve this workflow?
   2. Does radiology availability affect your ultrasound use? E.g., Do you find yourself more likely to perform a POCUS exam on nights/weekends when the ultrasound tech must come in from home? What about when radiology reads are done remotely and delayed?
5. **Overall, which barriers are the most important to address that would make using ultrasound more accessible or doable for you?**
6. **Next section: *We are looking to identify facilitators that could make POCUS use in the ED easier and more accessible*. (Inner setting)**
7. **POCUS knowledge and skills:**
   1. Do you feel that you would benefit from more educational resources or sessions, and if so, which ones?
8. **Archiving (and billing) processes:**
9. Would the implementation of a standardized documentation system (e.g. saving ultrasound images on the machine and documenting findings in your notes) facilitate your ultrasound use? Would it hinder it in any way? What about if there was a policy from the national VA?
10. How would having image synchronization into the electronic health record with viewability by other hospital providers affect your ultrasound use?
11. **Clinical image review and quality assurance feedback to users:**
12. How would having an internal review/QA process with individualized image feedback affect your ultrasound use? Would you feel more comfortable? Would you use it more or less?
13. Would having a faculty credentialing system in place affect your ultrasound use? How so?
14. ***The following questions pertain to your ED environment.* (Outer setting)**
15. Does other providers’ enthusiasm for ultrasound use and teaching impact your willingness to learn or practice POCUS? How could your clinical practice be influenced if your colleagues are using ultrasound more readily?
16. Do you feel that the ultrasound-trained faculty are supportive of ultrasound education and use in the department? What changes have they made that are useful? Not useful? What else could be done for future improvement?
17. Do you feel that ED leadership supports ultrasound use in the emergency department? What about *[name redacted]* hospital leadership? (Note that current POCUS users include providers in EM, hospital medicine, anesthesia/ICU, and pain medicine.)

***Finally, we will ask some questions regarding the past year’s ultrasound training interventions.* (Intervention characteristics)**

1. Did you participate in the ultrasound small group sessions in February/March 2022?
2. Did you participate in the large-group ultrasound introductory course in May 2022?
3. What did you like best from the ultrasound teaching sessions? What did you think went well?
4. What were the strengths and weaknesses of training with providers of diverse training backgrounds and varying levels of prior ultrasound experience?
5. Do you feel that participating in an interprofessional group environment facilitates your learning of ultrasound skills? Does it benefit or hinder you in any way?
6. **What did you think could be improved from the ultrasound teaching sessions?**
7. **What type of future sessions would be most useful for you?**
   1. Would you like to see traditional lecture format didactics with a PowerPoint presentation showing US images?
   2. A 5-minute US-specific topic during faculty meeting?
   3. Self-directed learning with online platforms such as Core Ultrasound?
   4. A useful link via email once a month to view an exam type on your own time?
   5. Hands-on training sessions in a large group?
   6. Hands-on training sessions in a small group?
   7. Would you consider participating in bimonthly QA image review sessions? How could this be useful?
   8. What about collaborative POCUS sessions between *[name redacted]* departments (EM/IM/ICU)?
8. In conclusion, do you have any additional comments or feedback?

*Thank you for your time and participation. This concludes the interview*.
