# Supplementary material for: A qualitative study of perceived barriers and facilitators to point-of-care ultrasound use among Veterans Affairs Emergency Department providers
Source: PLoS One. 2024 Nov 7;19(11):e0310404. doi: 10.1371/journal.pone.0310404 (PMC11542812; doi:10.1371/journal.pone.0310404)
Supplement: S1 Table — (DOCX) [file pone.0310404.s002.docx]

VA POCUS implementation study codebook

Nodes\\Convenience and efficiency

| Name | Description | Files | References |
| --- | --- | --- | --- |
| Academic vs. community hospitals | POCUS use variability in academic vs community clinical sites | 7 | 9 |
| Barrier & Facilitator- Ease of documentation | How easy it is to document a point-of-care ultrasound | 12 | 30 |
| Barrier & facilitator- Necessity and utility | Utility and necessity of using ultrasound based on the clinical site's patient population | 13 | 21 |
| Barrier- Alternative means of obtaining imaging | Availability of alternative imaging modalities and its effect on POCUS use | 11 | 14 |
| Barrier- Time constraints and limitations | Time required to perform the ultrasound | 14 | 41 |
| Barrier- Ultrasound operability | how hard or easy it is to operate the machine | 5 | 10 |
| Effects of radiology ultrasound technician availability on POCUS use | POCUS use when radiology is not available on nights/weekends and when providers must wait for teleradiology reports | 14 | 26 |
| Eliminating unnecessary or cumbersome steps | Perceived problems with having a complicated documentation process with multiple systems/steps | 11 | 31 |
| Perceived roles and responsibilities | Beliefs of who is responsible for procedures such as IV placement, central lines, bladder scans, etc. at the clinical setting (e.g. nurses, residents, attendings) | 8 | 11 |
| POCUS facilitators-archiving system | Perceived interventions that may facilitate POCUS use in the emergency department | 12 | 22 |
| POCUS facilitators-credentialing system | Existence of a faculty credentialing system | 14 | 17 |
| POCUS facilitators-equipment & resources | Availability of adequate ultrasound equipment (machines) and image quality | 5 | 11 |
| POCUS facilitators-quality assurance feedback | Ultrasound image review/quality assurance feedback process | 14 | 30 |
| Setting dependent policy changes | National VA policy or faculty credentialing system and how this coiuld impact POCUS use | 12 | 20 |

Nodes\\ED environment (space and place)

| Name | Description | Files | References |
| --- | --- | --- | --- |
| Barrier & Facilitator- Location, cleanliness, and maintenance of equipment | Ease of locating the ultrasound machines, whether they are clean or not, and whether they are in good maintenance | 12 | 26 |
| Facilitator- US machine availability and accessibility of ED resources | POCUS machine and equipment availability in the ED when working clinically | 14 | 24 |
| POCUS archiving system | POCUS archiving system existence and use | 10 | 26 |
| US machine usability and familiarity | Ease of ultrasound machine use and familiarity with the keys and platform | 14 | 39 |

Nodes\\Opinions on clinical use of POCUS in the ED (medical decision making)

| Name | Description | Files | References |
| --- | --- | --- | --- |
| Facilitator- Answering pointed questions | Using POCUS to help answer specific clinical questions | 9 | 16 |
| Facilitator- Assessing progression of a specific condition or pathology | Serial POCUS exams and its effect on evolving patient care | 5 | 8 |
| Facilitator- Augmenting the physical exam | POCUS in relation to the physical exam and how it can augment it | 11 | 16 |
| Facilitator- Coming up with differentials and diagnoses | POCUS's effect on differential diagnosis formation | 11 | 26 |
| Facilitator- Continuity of care | Shared POCUS image viewing across the hospital system as a potential facilitatory for POCUS use in achieving more continuous patient care | 2 | 2 |
| Facilitator- Determining need for further imaging and next steps | POCUS use in clinical decision making | 6 | 16 |
| Facilitator- Ensuring patient safety and satisfaction | Ultrasound's effect on patient safety and satisfaction | 7 | 8 |
| Facilitator- Standard of care | Participants' perceptions on how ultrasound is incorporated into medical practice standard of care | 10 | 11 |
| POCUS clinical utility- patient-centered care improvements | POCUS's effect on improving patient care efficiency and potentially satisfaction | 8 | 12 |
| POCUS clinical utility- provider improvements | Exams for which ED providers believe POCUS is useful in ED clinical practice | 10 | 24 |
| POCUS learners & resident teaching | POCUS learners & its use in resident teaching | 14 | 41 |
| POCUS use for ED procedures | Procedures for which ED providers believe POCUS is useful | 14 | 32 |

Nodes\\Opinions on POCUS education

| Name | Description | Files | References |
| --- | --- | --- | --- |
| Barrier- Variability of presentation | Differences and variabilities in patient presentation as potential barrier to POCUS use | 4 | 8 |
| Barrier and facilitator- Desire for more practice | Advanced training and skills that participants would like more practice with and believe could improve their clinical practice | 13 | 56 |
| Facilitator- Repetition | Practice and repetition of POCUS as potential facilitator for its use and skill retention/reinforcement | 12 | 29 |
| Participants' comfort with POCUS image interpretation | Participants' comfort with POCUS image interpretation | 14 | 21 |
| POCUS education and retention of skills | Participants' opinions on current educational POCUS sessions and skill retention | 11 | 33 |
| Trust & confidence in own POCUS knowledge and skills | Participants' confidence of own POCUS skills and its effect on clinical POCUS use and teaching | 14 | 58 |

Nodes\\Peer Influences, Feedback, and Teaching

| Name | Description | Files | References |
| --- | --- | --- | --- |
| Contagiousness of enthusiasm among colleagues | Colleagues' enthusiasm for POCUS as potential facilitator for its use | 14 | 17 |
| Facilitator- Guidance and teaching from experts in the field | Ultrasound faculty support, guidance, and teaching of POCUS | 13 | 36 |
| Future educational interventions | Participants' opinions on future educational POCUS sessions | 14 | 70 |
| Hospital-wide POCUS collaboration and communication | POCUS collaborative efforts and communication within the hospital | 14 | 32 |
| Opinions on ED leadership support | Participants' opinions on ED leadership support for POCUS training and use | 14 | 14 |
| Opinions on support from ultrasound faculty | Participants' opinions on ultrasound faculty enthusiasm and support for teaching/training providers on POCUS use | 14 | 22 |
| Opinions on VA hospital support | Participants' opinions on VA hospital leadership support for POCUS training and use | 14 | 16 |
| Perceptions of other ED providers' use of POCUS and peer influence | Perceived influence of other ED providers' use of POCUS during their clinical shifts on participants | 13 | 27 |
| POCUS group training- ultrasound workshops | Perceptions of training with providers of varying skills and effect on ultrasound education | 14 | 49 |
| Ultrasound Workshops- Hands on rather than didactic learning | Different teaching style preferences for POCUS education and perceived learning effectiveness by participants | 14 | 45 |
| Ultrasound Workshops- Practicing on real people rather than models | Advantages/disadvantages of using simulation with models vs healthy human subjects for POCUS learning | 8 | 12 |
| Ultrasound Workshops- Utility of predetermined patients | Ability to scan patients with known pathology vs healthy subjects with normal anatomy | 2 | 2 |
