# Supplementary material for: A qualitative study of perceived barriers and facilitators to point-of-care ultrasound use among Veterans Affairs Emergency Department providers
Source: PLoS One. 2024 Nov 7;19(11):e0310404. doi: 10.1371/journal.pone.0310404 (PMC11542812; doi:10.1371/journal.pone.0310404)
Supplement: Attachment — Submitted filename: Response to reviewers - PLOS ONE VA POCUS Aug 8.2024.docx [file pone.0310404.s004.docx]

PONE-D-24-03510 Reviewer comments August 7.2024

A qualitative study of perceived barriers and facilitators to point-of-care ultrasound use among Veterans Affairs Emergency Department providers

PLOS ONE

Dear Dr. Theophanous,

Thank you for submitting your manuscript to PLOS ONE. After careful consideration, we feel that it has merit but does not fully meet PLOS ONE’s publication criteria as it currently stands. Therefore, we invite you to submit a revised version of the manuscript that addresses the points raised during the review process.

Please address a minor concern described below.

Please submit your revised manuscript by Sep 21 2024 11:59PM. If you will need more time than this to complete your revisions, please reply to this message or contact the journal office at [plosone@plos.org](mailto:plosone@plos.org). When you're ready to submit your revision, log on to https://www.editorialmanager.com/pone/ and select the 'Submissions Needing Revision' folder to locate your manuscript file.

Please include the following items when submitting your revised manuscript:

- A rebuttal letter that responds to each point raised by the academic editor and reviewer(s). You should upload this letter as a separate file labeled 'Response to Reviewers'.
- A marked-up copy of your manuscript that highlights changes made to the original version. You should upload this as a separate file labeled 'Revised Manuscript with Track Changes'.
- An unmarked version of your revised paper without tracked changes. You should upload this as a separate file labeled 'Manuscript'.

If you would like to make changes to your financial disclosure, please include your updated statement in your cover letter. Guidelines for resubmitting your figure files are available below the reviewer comments at the end of this letter.

If applicable, we recommend that you deposit your laboratory protocols in protocols.io to enhance the reproducibility of your results. Protocols.io assigns your protocol its own identifier (DOI) so that it can be cited independently in the future. For instructions see: [https://journals.plos.org/plosone/s/submission-guidelines#loc-laboratory-protocols](https://nam11.safelinks.protection.outlook.com/?url=https%3A%2F%2Fjournals.plos.org%2Fplosone%2Fs%2Fsubmission-guidelines%23loc-laboratory-protocols&data=05%7C02%7Crebecca.theophanous%40duke.edu%7C9db853a8ff654c3136ba08dcb6f29089%7Ccb72c54e4a314d9eb14a1ea36dfac94c%7C0%7C0%7C638586400142797806%7CUnknown%7CTWFpbGZsb3d8eyJWIjoiMC4wLjAwMDAiLCJQIjoiV2luMzIiLCJBTiI6Ik1haWwiLCJXVCI6Mn0%3D%7C0%7C%7C%7C&sdata=qxCFUh8v9ZXL%2FTGMY4Sz1Hz4SEGDMN9uzSOCaCBK28Y%3D&reserved=0). Additionally, PLOS ONE offers an option for publishing peer-reviewed Lab Protocol articles, which describe protocols hosted on protocols.io. Read more information on sharing protocols at [https://plos.org/protocols?utm_medium=editorial-email&utm_source=authorletters&utm_campaign=protocols](https://nam11.safelinks.protection.outlook.com/?url=https%3A%2F%2Fplos.org%2Fprotocols%3Futm_medium%3Deditorial-email%26utm_source%3Dauthorletters%26utm_campaign%3Dprotocols&data=05%7C02%7Crebecca.theophanous%40duke.edu%7C9db853a8ff654c3136ba08dcb6f29089%7Ccb72c54e4a314d9eb14a1ea36dfac94c%7C0%7C0%7C638586400142808204%7CUnknown%7CTWFpbGZsb3d8eyJWIjoiMC4wLjAwMDAiLCJQIjoiV2luMzIiLCJBTiI6Ik1haWwiLCJXVCI6Mn0%3D%7C0%7C%7C%7C&sdata=3ywWFnjQJWqyf%2B0XVD9Q1s8q80Mpkt1u1S%2BwsAFFgew%3D&reserved=0).

We look forward to receiving your revised manuscript.

Kind regards,

Takeru Abe, Ph.D
Academic Editor
PLOS ONE

Journal requirements:

When submitting your revision, we need you to address these additional requirements.

1. Please ensure that your manuscript meets PLOS ONE's style requirements, including those for file naming. The PLOS ONE style templates can be found at

<https://journals.plos.org/plosone/s/file?id=wjVg/PLOSOne_formatting_sample_main_body.pdf> and

<https://journals.plos.org/plosone/s/file?id=ba62/PLOSOne_formatting_sample_title_authors_affiliations.pdf>

-The manuscript and title page were reformatted per journal requirements.

2. In the ethics statement in the Methods, you have specified that verbal consent was obtained. Please provide additional details regarding how this consent was documented and witnessed, and state whether this was approved by the IRB. – Added two sentences to the methods section: “Our study was deemed exempt by the VA Institutional Review Board, and consent was waived by the IRB (1631300-4).” “Verbal consent was obtained by reading aloud a written statement describing voluntary study participation at the beginning of each audio recorded interview and documented using a data collection checklist.”

3. Thank you for stating the following financial disclosure:

“RGT received a Society of Academic Emergency Medicine Foundation/Academic Emergency Ultrasound Section grant #AG 2020-0000000099. [https://www.saem.org/about-saem/academies-interest-groups-affiliates2/saem-foundation/apply-for-a-grant/academy-grants/aeus-research-grant.”](https://www.saem.org/about-saem/academies-interest-groups-affiliates2/saem-foundation/apply-for-a-grant/academy-grants/aeus-research-grant.%E2%80%9D)

Please state what role the funders took in the study.  If the funders had no role, please state: "The funders had no role in study design, data collection and analysis, decision to publish, or preparation of the manuscript." – This statement was added to the cover letter.

If this statement is not correct you must amend it as needed.

Please include this amended Role of Funder statement in your cover letter; we will change the online submission form on your behalf.

4. Thank you for stating the following in the Acknowledgments Section of your manuscript:

“This study was funded by an SAEMF-AEUS grant #AG 2020-0000000099.”

We note that you have provided funding information that is currently declared in your Funding Statement. However, funding information should not appear in the Acknowledgments section or other areas of your manuscript. We will only publish funding information present in the Funding Statement section of the online submission form.

Please remove any funding-related text from the manuscript and let us know how you would like to update your Funding Statement. Currently, your Funding Statement reads as follows:

“RGT received a Society of Academic Emergency Medicine Foundation/Academic Emergency Ultrasound Section grant #AG 2020-0000000099. [https://www.saem.org/about-saem/academies-interest-groups-affiliates2/saem-foundation/apply-for-a-grant/academy-grants/aeus-research-grant.”](https://www.saem.org/about-saem/academies-interest-groups-affiliates2/saem-foundation/apply-for-a-grant/academy-grants/aeus-research-grant.%E2%80%9D) – The funding statement is correct. The text was removed from the manuscript and added to the cover letter.

Please include your amended statements within your cover letter; we will change the online submission form on your behalf.

5. Please review your reference list to ensure that it is complete and correct. If you have cited papers that have been retracted, please include the rationale for doing so in the manuscript text, or remove these references and replace them with relevant current references. Any changes to the reference list should be mentioned in the rebuttal letter that accompanies your revised manuscript. If you need to cite a retracted article, indicate the article’s retracted status in the References list and also include a citation and full reference for the retraction notice. – References were reviewed and outdated website links updated.

[Note: HTML markup is below. Please do not edit.]

Reviewers' comments:

Reviewer's Responses to Questions

**Comments to the Author**

1. Is the manuscript technically sound, and do the data support the conclusions?

The manuscript must describe a technically sound piece of scientific research with data that supports the conclusions. Experiments must have been conducted rigorously, with appropriate controls, replication, and sample sizes. The conclusions must be drawn appropriately based on the data presented.

Reviewer #1: Yes

2. Has the statistical analysis been performed appropriately and rigorously?

Reviewer #1: N/A

3. Have the authors made all data underlying the findings in their manuscript fully available?

The [PLOS Data policy](https://nam11.safelinks.protection.outlook.com/?url=http%3A%2F%2Fwww.plosone.org%2Fstatic%2Fpolicies.action%23sharing&data=05%7C02%7Crebecca.theophanous%40duke.edu%7C9db853a8ff654c3136ba08dcb6f29089%7Ccb72c54e4a314d9eb14a1ea36dfac94c%7C0%7C0%7C638586400142817625%7CUnknown%7CTWFpbGZsb3d8eyJWIjoiMC4wLjAwMDAiLCJQIjoiV2luMzIiLCJBTiI6Ik1haWwiLCJXVCI6Mn0%3D%7C0%7C%7C%7C&sdata=OeO6AoDgXK3FRPUTP2yEWMeAomH6xMnOHHxoJiTWzaE%3D&reserved=0) requires authors to make all data underlying the findings described in their manuscript fully available without restriction, with rare exception (please refer to the Data Availability Statement in the manuscript PDF file). The data should be provided as part of the manuscript or its supporting information, or deposited to a public repository. For example, in addition to summary statistics, the data points behind means, medians and variance measures should be available. If there are restrictions on publicly sharing data—e.g. participant privacy or use of data from a third party—those must be specified.

Reviewer #1: Yes

4. Is the manuscript presented in an intelligible fashion and written in standard English?

PLOS ONE does not copyedit accepted manuscripts, so the language in submitted articles must be clear, correct, and unambiguous. Any typographical or grammatical errors should be corrected at revision, so please note any specific errors here.

Reviewer #1: Yes

5. Review Comments to the Author

Please use the space provided to explain your answers to the questions above. You may also include additional comments for the author, including concerns about dual publication, research ethics, or publication ethics. (Please upload your review as an attachment if it exceeds 20,000 characters)

Reviewer #1: Thank you for giving an opportunity to review this interesting work.
The authors described a qualitative study regarding barriers and facilitators to POCUS at EM department of VA Hospital. They presented data with sound methodologies.
This reviewer has a minor suggestion; it would be fruitful for readers if the authors add a description and an explanation regarding which analyses they adopted and did not adopt, such as content analysis with grounded theory and thematic analysis.
This information could help readers to understand your methodology better, so that the readers will be able to judge the findings' generalizability. -Details were added to the methods section about the analysis methodologies used as suggested by the reviewers.

6. PLOS authors have the option to publish the peer review history of their article ([what does this mean?](https://nam11.safelinks.protection.outlook.com/?url=https%3A%2F%2Fjournals.plos.org%2Fplosone%2Fs%2Feditorial-and-peer-review-process%23loc-peer-review-history&data=05%7C02%7Crebecca.theophanous%40duke.edu%7C9db853a8ff654c3136ba08dcb6f29089%7Ccb72c54e4a314d9eb14a1ea36dfac94c%7C0%7C0%7C638586400142826064%7CUnknown%7CTWFpbGZsb3d8eyJWIjoiMC4wLjAwMDAiLCJQIjoiV2luMzIiLCJBTiI6Ik1haWwiLCJXVCI6Mn0%3D%7C0%7C%7C%7C&sdata=PM0OG6bmRLuX%2Bsdy2ujRItuhIkKjxneqGWHKBN6yZRU%3D&reserved=0)). If published, this will include your full peer review and any attached files.

If you choose “no”, your identity will remain anonymous but your review may still be made public.

**Do you want your identity to be public for this peer review?** For information about this choice, including consent withdrawal, please see our [Privacy Policy](https://nam11.safelinks.protection.outlook.com/?url=https%3A%2F%2Fwww.plos.org%2Fprivacy-policy&data=05%7C02%7Crebecca.theophanous%40duke.edu%7C9db853a8ff654c3136ba08dcb6f29089%7Ccb72c54e4a314d9eb14a1ea36dfac94c%7C0%7C0%7C638586400142835841%7CUnknown%7CTWFpbGZsb3d8eyJWIjoiMC4wLjAwMDAiLCJQIjoiV2luMzIiLCJBTiI6Ik1haWwiLCJXVCI6Mn0%3D%7C0%7C%7C%7C&sdata=7hEYIaFuMVRi3jAY0LtKh9%2FLSO3vtz2dkpTAvi8Tk58%3D&reserved=0).

Reviewer #1: No

[NOTE: If reviewer comments were submitted as an attachment file, they will be attached to this email and accessible via the submission site. Please log into your account, locate the manuscript record, and check for the action link "View Attachments". If this link does not appear, there are no attachment files.]

While revising your submission, please upload your figure files to the Preflight Analysis and Conversion Engine (PACE) digital diagnostic tool, [https://pacev2.apexcovantage.com/](https://nam11.safelinks.protection.outlook.com/?url=https%3A%2F%2Fpacev2.apexcovantage.com%2F&data=05%7C02%7Crebecca.theophanous%40duke.edu%7C9db853a8ff654c3136ba08dcb6f29089%7Ccb72c54e4a314d9eb14a1ea36dfac94c%7C0%7C0%7C638586400142843022%7CUnknown%7CTWFpbGZsb3d8eyJWIjoiMC4wLjAwMDAiLCJQIjoiV2luMzIiLCJBTiI6Ik1haWwiLCJXVCI6Mn0%3D%7C0%7C%7C%7C&sdata=xd9Zg4GVBYPNEFhknRbcvvIv1bShy3%2BFwI7uHxZSMBk%3D&reserved=0). PACE helps ensure that figures meet PLOS requirements. To use PACE, you must first register as a user. Registration is free. Then, login and navigate to the UPLOAD tab, where you will find detailed instructions on how to use the tool. If you encounter any issues or have any questions when using PACE, please email PLOS at [figures@plos.org](mailto:figures@plos.org). Please note that Supporting Information files do not need this step.

*In compliance with data protection regulations, you may request that we remove your personal registration details at any time.* [*(Remove my information/details)*](https://nam11.safelinks.protection.outlook.com/?url=https%3A%2F%2Fwww.editorialmanager.com%2Fpone%2Flogin.asp%3Fa%3Dr&data=05%7C02%7Crebecca.theophanous%40duke.edu%7C9db853a8ff654c3136ba08dcb6f29089%7Ccb72c54e4a314d9eb14a1ea36dfac94c%7C0%7C0%7C638586400142849400%7CUnknown%7CTWFpbGZsb3d8eyJWIjoiMC4wLjAwMDAiLCJQIjoiV2luMzIiLCJBTiI6Ik1haWwiLCJXVCI6Mn0%3D%7C0%7C%7C%7C&sdata=LzzCZquphgnSki%2FxebALlaqO4VP28rUqy8zzeH7q3aI%3D&reserved=0)*. Please contact the publication office if you have any questions.*
